# The Week's Work

**Published:** 1911-07-22

**Authors:** 


### THE WEEK'S WORK.

### THE DANGER TO THE VOLUNTARY HOSPITALS.

ALL friends of the voluntary hospitals in this country must be disappointed with the barrenness and indeterminate phraseology of the memorandum regarding the National Insurance Bill which has just been issued by the King's Fund, and which we publish on another page. The King's Fund ought to be the supreme authority on hospital questions. Its opinion deservedly carries great weight with the public, and a vacillating, uncertain expression of opinion coming from it will influence the public to believe that there is a want of consensus among hospital workers concerning the effects of the Insurance Bill. It is imperative that this belief should be dissipated, and it is imperative too that the King's Fund, as representing the London hospitals, should consider the whole question of the Insurance Bill carefully and in the utmost detail, and publish a full and detailed memorandum setting forth the statistics of hospital revenue and the probable effects which the Bill in its present form will have, not only upon the hospitals, but upon the great funds as well. The present embryo memorandum satisfies nobody. It is a weak, uncertain, wavering note, which should be strengthened as soon as possible.

### THE POSITION AT ST. JOHN'S HOSPITAL.

WE understand that the newspaper statements concerning the alleged resignation of the medical staff of the St. John's Hospital for Diseases of the Skin are incorrect. We have pleasure in making this known, as the Board fears that the position of the hospital may be injured by the unauthorised statements that have been appearing in the lay press. At present the Board is reserving information, but we hope shortly to be able to publish the hospital's version of the case. In the meantime, it may be well to remind the public that St. John's Hospital is a pioneer institution in some respects. For example, its in—and out—patient departments are separated, the latter in Leicester Square being, of course, in the very heart of London, while the ward cases are treated at the Hammersmith branch. The bold course is generally the safe one for public institutions, and we shall be glad to publish the hospital's statement, which alone can check the ill-considered opinions that are still in circulation.

### KING EDWARD HOSPITAL, EALING, OPENED.

LAST Saturday the King Edward Memorial Hospital at Mattock Lane, Ealing, of which Messrs. Hall-Jones and Cummings are the architects, was opened by Princess Christian of Schleswig-Holstein. The hospital's President, Lord George Hamilton, received her Royal Highness, to whom Alderman Box, chairman of the Building Committee, and Mr. Beaumont Shepheard, J.P., treasurer, Mr. Hedges, Mr Hetherington, Mr. Chambers, Mr. Kimmitt, hon. secretaries, Dr. Hogg, Dr. Lyle, Mr. Charles Jones, and Mr. J. W. Bailey were presented. The hospital, with its present accommodation of forty beds, and with its possibility of extending that number to ninety, has the enviable, if rare, distinction of being most attractive in appearance, for which the architects deserve our gratitude. You enter a small octagonal hall, round which the administration rooms are grouped, while overhead are two floors of nurses' bedrooms. Passing through the hall to the back you enter a quadrangle, the east side of which is a ward pavilion of two storeys, the south and north sides being formed by a covered corridor with semi-open sides, the centre of the open square being occupied by the theatre. Beyond this again is an open space which can be built on later. To the extreme right is the dispensary, which has been active for two years, and from which the present hospital has grown. The move from the old hospital will be made within a fortnight, and we understand negotiations are now in progress for its sale. The gilt tablet in the hall, recording the foundation of the hospital, is the work of Mr. F. Bowcher. Mr. William Hedges, for so long the honorary secretary, must feel proud of his new institution, which the Ealing public has supported so well.

### A "LUCAS" BED FOR UNIVERSITY COLLEGE HOSPITAL.

THE life-work of the late Mr. Henry Lucas, whose achievements as Chairman and Treasurer of University College Hospital were recorded in THE HOSPITAL of November 12, 1910 (page 206), has now been commemorated by the gift of £1,000 from Mrs. Lucas and her children to endow a bed in memory of him. Captain the Hon. E. S. Dawson, R.N., his successor in the treasurership, and the whole hospital staff will feel that Mrs. Lucas could have chosen no apter memorial.

### A MEDICO MECHANICAL INSTITUTE FOR SHEFFIELD.

THE first medico-mechanical institute in England will be founded shortly in Sheffield through the gift which Mr. Edgar Allen has presented to the city for that purpose. It is stated that Mr. Allen has had the institute fitted with a complete set of Zander apparatus, and that he will bear the whole cost of maintenance, £1,500 a year, for the next three years. By that time it is hoped the institute will be supported in conjunction with the hospitals with which it will be worked. The institute, we understand, is intended for the free treatment of wage-earners suffering from limb affections. It is recalled that Mr. Allen has already given £20,000 for other purposes to the city.

### A RECORD COLLECTION IN BIRMINGHAM

ALDERMAN SIR HALLEWELL ROGERS, president, and Mr. W. S. Aston, the honorary secretary, of the Birmingham Hospital Saturday Fund, are to be congratulated on having achieved a record collection this year. A few months ago £21,000 was asked for, and by the time this issue is in the hands of our readers it is safe to prophesy that this sum will have been passed. At the moment of writing over £20,000 has been received, and the fund stands already in the proud position of being £600 in advance of the total subscribed last year, and of having beaten all previous records by over £200. It is highly satisfactory to note that this sum has been raised in spite of the introduction of the Government's insurance proposals, and it is a useful sign of the strength of the voluntary system in the year preceding the working of any Act based upon them.

### THE LIABILITY OF HOSPITAL SURGEONS FOR NEGLIGENCE.

An action has recently been brought against the Assistance Publique and a hospital surgeon for £2,000 damages on account of injuries received in the course of an operation for appendicitis upon a patient admitted to the Bichat Hospital. The surgeon, while the patient was under an anæsthetic, made use of the thermocautery, and unfortunately the alcohol employed to heat the needle caught fire, with the result that the patient was so badly burned in various parts of the body as to necessitate his detention in the hospital for four months. When the action came on for trial the tribunal acquitted the Assistance Publique of responsibility on the ground that the surgeon could not be held to be an employee of that department. The principle of the responsibility of the surgeon was, however, agreed to provided that it could be shown that he was guilty of negligence. The tribunal therefore appointed three experts well known in the French surgical world to determine the limits within which the surgeon could be held to have committed a professional fault, and the case was accordingly adjourned to await their report. It will be interesting to await the outcome of the experts' decision. In any event the accused surgeon is fortunate in having his conduct judged by his peers, and in not having to rely for justice on the ignorant prejudices of a lay jury in a purely professional matter.

### THE WATSON BEQUEST.

The Nottingham General Hospital has been left £10,000 by Mrs. Elizabeth Anne Watson, an inhabitant of the city, the income of which is to be applied "in providing accommodation for as many in-patients as such income shall suffice for." It is not often that a hospital has ten beds endowed at one time, for this is to what the Watson bequest amounts, and the fact is worth noting. The phrasing of the clause above quoted, however, seems to us a little ambiguous. "Providing accommodation" might be interpreted in a structural rather than in a maintenance sense, though that, probably, would not be the sense in which the testator wished the clause to be interpreted. In any case the application of the income from the £10,000 will be awaited with some interest. Mrs. Watson's will was elaborate in its bequests to charities and to her servants, and Mr. Erasmus Keely, the secretary, and Mr. B. A. Smith, the treasurer, are to be congratulated on the sum about to be placed at their disposal.

### RENOVATION OF THE HOTEL DIEU AT PARIS.

The Assistance Publique proposes to devote a portion of the funds resulting to it from a municipal loan of 900 million francs to transforming the Hôtel Dieu. The essential innovation contemplated consists in the installation of a maternity department in the hospital, a department which has been lacking since the demolition of the annexe in the rue de la Bûcherie. In addition it is proposed to unite under one roof the various ophthalmological departments which at present are scattered throughout the hospital, as well as to build a new laundry and two new operating theatres with up-to-date equipment. The majority of other special departments will be installed anew under better conditions, and a department of radiography will be provided so as to allow of patients being examined in the hospital itself. Electric lighting and steam-heating is to be installed throughout. It is calculated that the cost of these alterations will amount to 1,810,000 francs or £72,400.

### THIS WEEK'S DRUG MARKET.

Business in the drug market has been rather quiet during the week, with few price changes of any note. The article which has attracted the greatest attention is quicksilver, which has again advanced in price; makers of calomel, corrosive sublimate, and other salts of mercury have raised their quotations in consequence. The price of opium is maintained, but there has been no further advance, and the excitement which recently prevailed in this market has subsided; morphine and codeine are unchanged; apomorphine is dearer. Buchu leaves and ipecacuanha are, if anything, rather lower in price. Essence of lemon is again somewhat dearer. Extreme prices were paid for asafœtida at the recent drug auctions.

